# Cincinnati Dental Association

**Published:** 1862-03

**Authors:** 


### CINCINNATI DENTAL ASSOCIATION.

This Society has been holding regular semi-monthly meetings for the past three or four months, and though not largely attended, they have been very interesting and profitable to those engaged.

The study of the microscopic structure of the teeth has occupied considerable time, and all feel profited by their efforts in this direction. Essays and discussions occupy a due share of the time.

A new feature has been added, viz : the appointment at each meeting of a committee of one, denominated the committee of improvement, whose work it is to bring before the Society something new, either in instruments, practice, or improvements. In this way things are brought out that would have otherwise remained unknown.

We ought, perhaps, to have made reports of the proceedings, but many of our brethren of the city and vicinity have, we suppose from a want of interest, neglected to meet with us; and the presumption is that the reports, at least to them, and it may be to others, would be uninteresting and "common place." Our local Society will *live*, and operate much benefit to its active members.

